# Deep vein thrombosis inhibitor may play a therapeutic role in post-stroke patients

**DOI:** 10.1186/s12881-020-01108-9

**Published:** 2020-10-22

**Authors:** Xixi Xiang, Di Yuan, Peiyan Kong, Ting Chen, Han Yao, Shijia Lin, Xi Zhang, Hongbao Cao

**Affiliations:** 1 State Key Laboratory of Trauma, Burns and Combined Injury; Medical Center of Hematology, The Second Affiliated Hospital of Army Medical University, Key Subject of Chongqing, No. 83 Xinqiao Street, Shapingba District, Chongqing, 400037 PR China; 2 Department of Educational Technology, College of Basic Medical Sciences, Army Medical University, Chongqing, 400038 China; 3 Department of Psychiatry, First Hospital/First Clinical Medical College of Shanxi Medical University, Taiyuan, 030001 Shanxi Province China; 4 School of Systems Biology, George Mason University, Fairfax, Virginia, 22030 USA; 5 Department of Genomics Research, RD Solutions, Elsevier Inc., Rockville, MD 20852 USA

**Keywords:** Deep vein thrombosis, Stroke, Mega-analysis, Pathway analysis, Multiple linear regression analysis

## Abstract

**Background:**

Deep vein thrombosis (DVT) is associated with stroke. Here, we hypothesize that genes associated with DVT may also play roles in the development of stroke.

**Methods:**

we firstly conducted large-scale literature based disease-gene relationship data analysis to explore the genes implicated with DVT and stroke. Further, a mega-analysis was conducted for each of these genes that were linked to DVT but not stroke, using 11 independent stroke RNA expression datasets (176 stroke cases and 102 healthy controls). Then, a multiple linear regression (MLR) model was employed to study possible influential factors on the gene expression levels in stroke. After that, a functional pathway analysis was performed to identify the potential biological linkage between stroke and the target genes suggested by mega-analysis.

**Results:**

Over 81.10% genes implicated with DVT also suggested an association with stroke. Among the 24 DVT-specific genes, one DVT-inhibiting gene, *SP1*, presented significantly increased expression in stroke (LFC = 1.34, *p*-value = 0.0045). Pathway analysis showed that *SP1* may play a therapeutic role in post-stroke patients by promoting multiple of stroke-inhibitors. Moreover, geographical region was indicated as an influential factor on the expression levels of *SP1* in stroke samples (*p*-value = 0.037).

**Conclusion:**

Our results suggested that DVT inhibitor *SP1* could be a novel therapeutic target gene for post-stroke treatment. Further study of the potential relations between *SP1* and stroke was guaranteed.

## Background

Deep vein thrombosis (DVT) has been well-known to be associated with stroke [1, 2], which is also supported by many clinical studies [3–5]. The genetic and genomic variants implicated in stroke patients and DVT susceptibilities may partially explain the association between DVT and stroke. For instance, the expression and activity of matrix metalloproteinase-9 (MMP9) were significantly increased during stroke in human [6] and were associated with the early phase of DVT resolution [7]. These previous findings have paved a solid foundation for the study of associations between stroke and DVT.

Previously, several studies indicated the possible connection between DVT and stroke. For example, researchers from Danish found a strong linkage between hospital admission for venous thromboembolism and stroke through a 20-year cohort study. They reported that patients having the DVT are more likely to get attacked by stroke, especially in the first year after having a DVT [8]. Here, we explore the relation between DVT and stroke at the genetic level, trying to identify novel common genes that associate with both diseases. We hypothesize that genes of DVT with increased activity or expression levels may also play roles for the etiological development of stroke.

## Methods

To test our hypothesis, we organized the workflow as follows. First, a large-scale literature-based mining effort for DVT- and stroke-gene sets was undertaken. Then, for each of the DVT-specific genes, a mega-analysis and was conducted with 11 publicly available stroke expression datasets retrieved from Gene Expression Omnibus (GEO) (https://www.ncbi.nlm.nih.gov/geo/). For these genes that showed significant expression change across analyzed datasets, a literature-based functional pathway analysis was conducted, then predictions on their pathogenic significance in stroke were made. In addition, a multiple linear regression (MLR) model was employed to study the possible influence of sample size, sample organism, population region, and study date on the gene expression levels in stroke.

### Large-scale literature based disease-gene relationship data analysis

Relation data for both DVT and stroke were extracted from existing literature and analyzed using Pathway Studio (www.pathwaystudio.com) and then were downloaded into a genetic database DVT_Stroke, hosted at http://database.gousinfo.com. The downloadable format of the database in excel is available at http://gousinfo.com/database/Data_Genetic/DVT_Stroke.xlsx. Besides the list of analyzed genes (DVT_Stroke→DVT_ genes, Stroke_ genes, and DVT_specific genes), supporting references for each disease-gene relation are presented at DVT_Stroke→DVT_relation and Stroke_relation, including titles of the references and the sentences describing identified disease-gene relationships. The information could be used to locate a detailed description of an association of a candidate gene with DVT and/or Stroke.

### Stroke RNA expression datasets selection for mega-analysis

All expression datasets were searched at GEO through a keyword ‘stroke’. Then, the following standards were applied to do the further filter: 1) The entry type is series; 2) The study type is RNA expression by array; and 3) the studies are performed according to case-control design.

To note, the selection of the data covers all stroke expression array datasets from GEO, which is owned by the National Institutes of Health (NIH of USA). The datasets are publicly available, and no permission or confirmation is needed from any individual investigators. Moreover, datasets extraction has no selection bias in terms of publication journals, owner affiliations, and authors. In addition, the original data rather than the processed results of each dataset were used to perform the analysis in this study, which makes the process a mega-analysis rather than meta-analysis to avoid possible noise caused by individual data process.

### Mega-analysis models

Both the fixed-effect model and random-effects model [9] were employed to study the effect size of DVT related genes in case of stroke. For each of the stroke-expression dataset, the log fold change (LFC) was calculated for the stroke samples and used as the index of effect size in mega-analysis. The expression data were normalized and log2-transformed if not done in the original dataset. Results from both models were reported and compared. The heterogeneity of the mega-analysis was analyzed to study the variance within and between different studies. In the case that the total variance Q is equal to or smaller than the expected between-study variance df, the statistic ISq = 100% x (Q-df)/Q will be set as 0, and a fixed-effect model was selected for the mega-analysis. Otherwise, a random-effects model was selected. The Q-p represents the probability that the total variance is coming from within-study only. All analysis was conducted by using MATLAB (R2017a) mega-analysis package.

Results from mega-analysis and significant genes were identified following the criteria: *p* < 0.005 and effect size (LFC) > 1 or < − 1 and presented in the DVT_Stroke→Mega-analysis. The discussion will be focused on the gene that satisfies the significant criteria.

### Multiple linear regression analysis for the risk factors on stroke

We applied a multiple linear regression analysis to study the possible influence of four factors on the gene expression change in stroke: sample size, sample organism, population region, and study age. *P*-values and 95% confidence interval (CI) were reported for each of the factors. The analysis was done in Matlab (R 2017a) with the ‘regress’ statistical analysis package.

### Functionality literature-based pathway analysis

A literature-based functional pathway analysis was conducted with an aim to identify the potential biological linkage between stroke and the target genes selected from mega-analysis. The pathway analysis was performed using the ‘Shortest Path’ module of Pathway Studio (www.pathwaystudio.com). A follow-up mega-analysis was conducted to validate the performance of the genes involved in this functional pathway.

## Results

### DVT-stroke implicated genes

Pathway Studio guided literature data-mining for the genes associated with DVT yielded 127 genes, while stroke was associated with 1220 genes. A significant overlap between DVT- and stroke-genes (103 genes; *p*-value = 2.04e-81), only 18.89% of the DVT-related genes (24 genes) have not been implicated in stroke. The full list of DVT- and stroke-genes, and 24 DVT alone genes are presented in DVT_Stroke→DVT_genes, stroke_genes, and DVT_specific genes.

### Mega-analysis results

As shown in Table 1, a total of 11 independent RNA expression datasets qualified the filter criteria were utilized for the mega-analysis (176 cases VS. 102 healthy controls), which were distributed in six different countries, three samples organisms, and study age ranged from 6 to 13 years.

**Table 1:** Datasets selection for mega-analysis

Study Name	Dataset GEOID	Sample size (control/case)	Country	Study Age	Sample Organism
Jickling et al.,2014	GSE21136	6/24	USA	6	*Rattus norvegicus*
White et al.,2014	GSE30655	6/14	USA	6	*Mus musculus*
Buga et al.,2014	GSE55260	2/4	Germany	6	Rattus norvegicus
Stamova et al.,2014	GSE58294	23/69	USA	6	*Homo sapiens*
Barreto et al.,2013	GSE28731	4/6	USA	7	Mus musculus
Liu et al.,2013	GSE46267	1/6	Singapore	7	Rattus norvegicus
Krug et al.,2011	GSE22255	20/20	Portugal	9	Homo sapiens
Mitsios et al.,2007	GSE9391	3/3	United Kingdom	13	Homo sapiens
Milbauer et al.,2007	GSE9877	27/20	USA	13	Homo sapiens
Wang et al.,2013	GSE38037	4/4	USA	7	Rattus norvegicus
Hori et al.,2012	GSE28201	2/2	Japan	8	Mus musculus

**Fig. 1:**
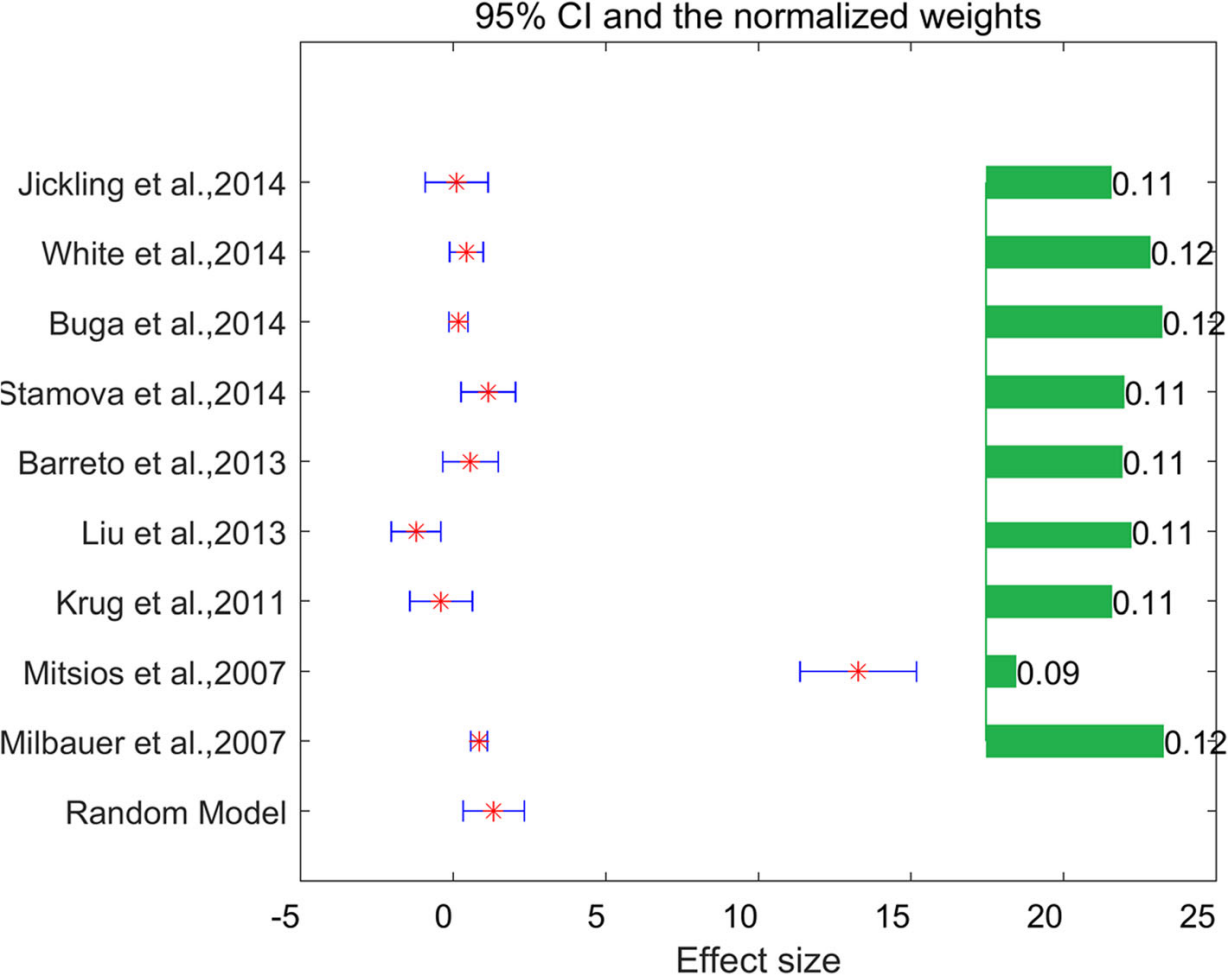
Mega-analysis results for in the case of stroke. The green bars on the right represent the weight of each datasets; the red stars on the left are the log fold change (LFC) of SP1 within each dataset; the width of the horizontal bar on the left represents the 95% confidence level of the corresponding LFC of SP1

For each of the 24 DVT-specific genes, a log fold change (LFC) was estimated from the majority of the 11 studies. However, only one gene, *SP1*, satisfied the significance criteria (*p* < 0.005 and effect size (LFC) > 1). Specifically, *SP1* presented significant increased LFC in case of stroke in mega-analysis. The effect sizes and related statistics results were presented in Table 2. Heterogeneity analysis results showed that, for *SP1*, there was significant between-study variance for mega-analysis (ISq = 96.1, *p*-value-Q < 0.001), and therefore, a random-effects model was selected (Fig. 1).

**Table 2:** Gene that passed the significant criteria for being involved in both DVT and stroke

Gene Name		***SP1***
**Mega-analysis**	Random Effects Model	Yes
	Datasets included	9
	LFC	1.34
	STD of LFC	0.51
	p-value	0.0045
	ISQ	96.11
	p-value-Q	< 0.001
**Multiple linear regression analysis**	Sample Size	0.96
	Population Region	0.037
	Sample Organism	0.095
	Study Age	0.12

### MLR analysis results

Results from the MLR models showed that population region (country) was a significant influencing factor for the expression fold change of *SP1* (*p*-value = 0.037), as shown in Table 2. Sample size and study age were not significant influential risk factors. It should also be noted that the sample organisms of the nine datasets were different, including *Rattus norvegicus*, *Mus musculus*, and *Homo sapiens* (Table 1). However, different sample organisms presented no significant impact on the expression fold change of *SP1* (p-value = 0.095).

### Literature-based pathway analysis results

**Fig. 2:**
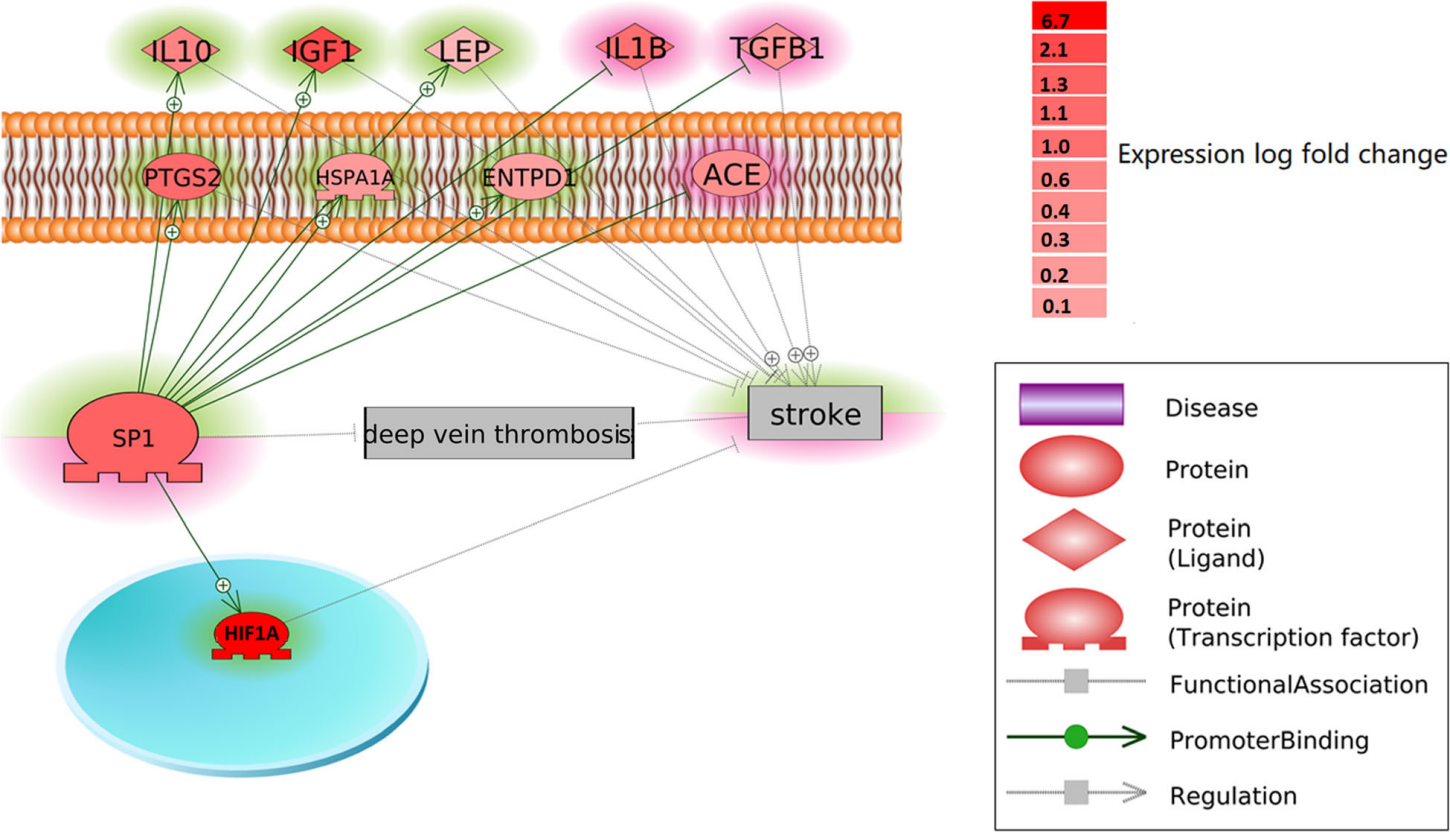
Functional pathway between stroke and gene *SP1*. The pathway was firstly built by using Pathway Studio, where the relations between any of the two entities were supported by one or more references. Then, a meta-analysis has been conducted for each of the genes presented in the pathway using the 12 datasets employed in this study. The genes are color-coded by their expression log fold change. The genes highlighted by green were supported by the meta-analysis results, while the red-highlighted ones were not

According to the previous large-scale literature-based approach selected for identification of novel stroke-related genes, no prior direct relations to the pathogenesis of stroke were known for *SP1*. However, Pathway Studio-guided the ‘shortest path’ revealed multiple pathways connecting SP1 and stroke (Fig. 2), which were supported by at least one reference (See DVT_Stroke→ShortestPath). The pathways indicated two potential mechanisms that SP1 may play a protective role against the development of stroke: 1) SP1 promotes stroke-inhibitors (see Fig. 2, highlighted by green); 2) SP1 inhibits stroke-promoters (see Fig. 2, highlighted by red).

To validate the pathways built using the ‘Shortest path’, we conducted a meta-analysis using the 12 datasets employed in this study, and encode the genes by color (see the color bar of Fig. 2). The detailed results of mega-analysis of these genes were presented in DVT_Stroke→ShortestPath_Mega. Results showed that the pathways where SP1 promoting stroke-inhibitors were activated, while the ones where SP1 inhibiting stroke-promoters pathways were not. For the detailed mega-analysis results of the genes in Fig. 2, including *p*-value and log fold change, please refer to DVT_Stroke➔SP1 promoting stroke-inhibitors.

## Discussion

Our study is a novel approach to identify the not-yet described molecular pathways which associate the process of DVT and the development of stroke. By removing all known intersections between curated genomes involved in each of these pathophysiological processes, we ensured that 24 DVT-specific genes to stroke had not been already described as such. Then, 11 stroke RNA array-expression datasets acquired from GEO (Table 1) were utilized to test the correlation between each of these 24 genes and stroke.

Results from mega-analysis showed that expression levels of eight DVT-alone genes were significantly changed in stroke-cases as compared to normal controls (p-value < 0.05, see in DVT_Stroke→Mega_Analysis). However, only one of eight DVT-alone genes, *SP1*, passed the pre-selected significance of association criteria (*p* < 0.005 and LFC > 1) (Table 2, Fig. 1). In specialty, LFC of *SP1* was 1.34 from mega-analysis, demonstrating the changes of *SP1* were increased by more than 150% (Table 2), suggesting it was a potential stroke biomarker and maybe possibly involved in the development of stroke. Although sample size, sample age, and sample organism of 11 datasets were used in the mega-analysis, the population region (country) was the only factor that could affect the expression of *SP1* in case of stroke (*p* = 0.037, Table 2).

As shown in Fig. 2 (a), *SP1* acts as a hub-gene constructing multiple potential pathways that could contribute to stroke based on “promoter binding” and “expression” levels. In order to confirm the pathways (edges) presented in Fig. 2, a further mega-analysis was used to test the performance of the pathway genes in Fig. 2, using the 11 datasets employed in this study (Table 1). Results showed that not all the pathways in Fig. 2 were supported by the 11 datasets, which was expected. The pathways built in Fig. 2 were literature-based, which integrated information from different modality of data with varied platforms. However, majority of the activity of the genes were confirmed from the expression datasets and strengthened the validity of the identified pathway. As shown in Fig. 2, *SP1* promotes 7 inhibitors of stroke, including *PTGS2, L10, IGF1, LEP, ENTPD1, HSPA1A* and *HIF1A.* However, the literature-based pathways also suggested that *SP1* could inhibit three stroke-promoters (*IL1B, TGFB1,* and *ACE*), which was not supported by the 11 datasets employed in this study (these genes demonstrated increased expression levels in the case of increased *SP1* expression). These results suggest that *SP1* is more likely to play a therapeutic role, rather than preventive role, in the pathological development of stroke.

The protein encoded by *SP1* is a zinc finger transcription factor that binds to GC-rich motifs of many promoters. It involves in many cellular processes, including cell differentiation, cell growth, apoptosis, immune responses, response to DNA damage, and chromatin remodeling. In principle, variations in promoter sequences can alter gene expression directly by altering a transcription factor binding site, and promoter variants with effects on the transcriptional activity of certain human genes have been identified as disease risk factors [10]. For instance, in the *SP1 → LEP→*stroke pathway, *SP1* binds in the promoter region for the *LEP* gene. As a result, altered *SP1* transcriptional activity leads to promoted production of leptin [11], which ameliorates neurological deficits and reduces infarct volumes after stroke [12]. In another pathway, *SP1 → IL10 →* stroke, *SP1* positively regulates the transcription of *IL10* [13]*,* which has been successfully used as a therapeutic mediator to reduce post-stroke secondary neuroin-flammation [14]. More pathways have been revealed in Fig. 2 with detailed presented in DVT_Stroke→ShortestPath. These pathways got support from the 11 datasets employed in this study. These results suggested the possible mechanisms through which *SP1* plays a post-stroke therapeutic role.

Although the discussion was mainly focused on the gene *SP1* that presented significant expression change in the case of stroke, other genes with minor expression variances may also worth a closer look, including PF4 (LFC: 0.79; *p*-value< 10–3), CYP4V2(LFC: 0.72; p-value = 0.046). Literature based pathway analysis suggested that these two genes may related to stroke through multiple pathways (see DVT_Stroke: PF4_CYP4V2). However, further study using experimental data are needed to validate these pathways. In addition, 8 out of the 24 DVT-specific genes were not included in the 11 stroke expression data collected in this study, and therefore were not reported in the mega-analysis (see DVT_Stroke: Mega_Analysis). Analysis with datasets including these genes are needed to explore their potential role in stroke.

## Conclusion

The presented results support the hypothesis that genes associated with DVT may also play roles in the etiological development of stroke. Enhanced activity of *SP1* may contribute to the therapeutic effect in post-stroke patients by promoting multiple of stroke inhibitors. Further investment is needed to test the conclusion of this study.

## Data Availability

All data supporting the findings of this study are available from the corresponding author in response to a reasonable request.

## Abbreviations

DVT: Deep vein thrombosis
GEO: Gene Expression Omnibus
MLR: multiple linear regression
LFC: log fold change
